# Harms and Health Inequalities in the Night-Time Economy: A Qualitative Exploration of Professional Stakeholders’ Experiences and Explanations

**DOI:** 10.1007/s11524-026-01108-9

**Published:** 2026-07-27

**Authors:** Paige M. Hulls, Michael P. Daly, Zoe L. Clarke, Katie Powell, Elizabeth McGill, Polly Reynolds, Frank de Vocht, Laura Fenton, Matt Egan, Cheryl McQuire

**Affiliations:** 1 Bristol Medical School, Population Health Sciences, University of Bristol, Canynge Hall, 39 Whatley Road, Bristol, UK; 2 Sheffield Centre for Health and Related Research (SCHARR), School of Medicine and Population Health, University of Sheffield, Sheffield, UK; 3 Department of Public Health, Environments and Society, London, School of Hygiene & Tropical Medicine , 15-17 Tavistock Place, London, UK; 4 Public Health, Camden Council, London, UK; 5 National Institute for Health Research Applied Research Collaboration West (NIHR ARC West), Bristol, UK

**Keywords:** Night-time economy, Health inequalities, Unhealthy commodities, Local authorities, Alcohol, Gambling

## Abstract

Goods and services consumed in the night-time economy (NTE) are associated with adverse health outcomes. These outcomes are more prevalent in areas with greater deprivation and among minoritized population subgroups. This study was aimed at exploring the views of local authority stakeholders on the harms and health inequalities in the NTE. We focused on inequalities from alcohol and gambling, their relationship with the NTE and how different forms of power might underpin the inequalities generated. We interviewed 17 local authority and third sector stakeholders from two case study areas (Swindon and Derby) who had a professional remit for the night-time economy. Data were analyzed using framework analysis using McCartney’s Sources of Power as a guiding framework for the analytical narrative. Participants highlighted the cumulative impact of years of economic decline in the NTE and resultant tension between local authorities’ public health and economic priorities. For instance, the economic benefits of alcohol licenses were felt to be prioritized over potential adverse health outcomes. Collaboration and the adoption of a whole-system approach were suggested as ways of reducing harms in the town centers and improving users’ perceived safety. However, frustration was expressed at the lack of flexibility in how funds could be spent and the need to give local authorities more power to allocate funds according to the area’s unique challenges. Stakeholders identified that different population sub-groups had varying experiences in using the NTE in their local authorities and consequently had different requirements for what the NTE should ideally offer. Participants highlighted a need for future NTE spaces to accommodate all members of the different communities it serves. This is one of the few qualitative studies that explores the wider contextual factors that underpin persistent inequalities in harms associated with unhealthy commodities. Our findings can be used to help policymakers inform their decision-making process to address these harms and inequalities.

## Introduction

The NTE (night-time economy, NTE) refers to the diverse social, cultural, and economic activity occurring during evening and night-time hours. While exact definitions differ, the NTE commonly covers a wide range of activity in town and city centers taking place between the hours of 6 pm and 6 am, which can include retail, culture and leisure, transport, and accommodation [1, 2]. Over the past two decades, the UK’s NTE has grown markedly, with consumer spending in the NTE amounting to £93.7 billion in 2021, approximately 7% of UK consumer spending, despite the impact of COVID-19 restrictions on the NTE [3]. NTE growth creates new social, employment, revenue, and investment opportunities for towns and cities as well as the general population, and in some places, it has been credited for regenerating neighborhoods [4–6]. However, the impact of COVID-19 and austerity continues to affect the NTE, and in 2025, there was a 4.1% net decline in the number of venues operating late at night in the UK. Consequently, the number of late-night venues (i.e., clubs, casinos, and late bars) in the UK has reduced by 28.2% since March 2020, when COVID-19 restrictions were first introduced.

Despite economic and social benefits, the NTE also creates conditions that can lead to health and social harms. These harms affect some population subgroups more than others. For example, people of lower socio-economic status are more likely to experience greater levels of alcohol-related harm compared to their wealthier counterparts, despite consuming the same or less alcohol, a phenomenon known as the alcohol-harm paradox [7]. However, the factors that lead to, and perpetuate, these persistent health inequalities remain poorly understood. Health inequalities are defined as “systematic, avoidable and unfair differences in health outcomes that can be observed between populations, between social groups within the same population” [8].

The sources of power framework were developed by McCartney et al. (8) to consider whether the concept of power is an essential element in maintaining, increasing or reducing social, economic and health inequalities (Dickie, 2015; McCartney et al. 8). Several elements of socioeconomic status (including relative income, wealth, and power) have been previously confirmed as having a persistent relationship with health outcomes and are considered key causes of health inequalities [9]. More recently, stigma [10] and racism [11] are acknowledged as fundamental causes of health inequalities. 

Goods and services consumed in the NTE, including alcohol and gambling activities, are associated with adverse health outcomes [4]. The NTE encourages heavy alcohol consumption and users of the NTE consume more alcohol than the UK national average [12]. Individuals considered “heavy drinkers” are disproportionately attracted to the NTE[13] (citation needed). Binge drinking, a term used to describe the consumption pattern of a given amount of alcohol on a single occasion, takes place within the NTE, commonly occurring at weekends, including among individuals who report no or low alcohol consumption during weekdays [13]. Binge drinking has been associated with acute and chronic harms to the individual (e.g., accidents and liver damage) and wider communities (e.g., crime and disorder and public nuisance) [14]. In the UK, binge drinking is defined as drinking six or more units for women or eight or more units for men in a single session [15].

Crimes that occur at night are more likely to involve alcohol than day-time crimes and areas where night-time violence is more prevalent tend to have more active NTEs [16]. In the UK, approximately 62% of night-time violence is alcohol-related [12]. Consequently, nearly half of the 30,000 respondents who took part in a public consultation survey on alcohol-related harms in the North West of England reported avoiding the town centers at night due to the drunken behavior of others[17]. Many young women report that sexual harassment is a common experience in the NTE [18]; prior research suggests overcrowding in bars and nightclubs can facilitate this by giving perpetrators a sense of anonymity [19, 20]. NTE activities also have wider social and environmental costs, including increased use of ambulances and hospitals, litter on the streets, and noise and light pollution [2].

Gambling is also common in the NTE. It has typically been framed by the industry as an issue of personal responsibility; however, the rise of social gambling has led to poorer health outcomes for specific groups [21]. Social gambling can be defined as the activity of playing a gambling-related game online on a social network or media platform [22]. Gambling at night leads to increased sleep disruption as well as significantly higher anxiety and depressive symptoms [23], and for online gamblers, experiencing sleep deprivation leads to negative financial outcomes and experiencing a loss of control [24]. Those who are unemployed and living in the most deprived areas are more likely to be classified as experiencing “problem gambling” [25]. Gambling at elevated levels of risk is also associated with increased alcohol consumption [26].

Gambling expenditure is positively correlated with socioeconomic status [27]and although higher income groups have higher rates of gambling, adverse outcomes are greater among lower-income groups [28]. One possible explanation is there are higher concentrations of gambling outlets in areas with greater socioeconomic disadvantage [29]. Research looking at the socio-spatial patterning of outlets which sell high fat, sugar, or salt (HFSS) foods, alcohol, and tobacco alongside gambling outlets, has found that these tend to cluster in more deprived areas [30–32]. This suggests a need for interventions that address the socioeconomic determinants of health in the NTE [33]. While persistent inequalities in harms related to the NTE and these unhealthy commodities are well known, less is understood about the factors that underpin these inequalities, particularly within complex local systems [30].

To develop effective interventions addressing the harms and inequalities associated with NTE activities, intervention developers must first identify mechanisms of change by determining the causal links between exposures and outcomes, as well as considering contextual factors [34]. This can be achieved by engaging with stakeholders and service users, to elicit and understand their priorities for the NTE and to understand the complex system within which the NTE operates [35, 36]. Intervention acceptability (to stakeholders and users) and system compatibility are key considerations for interventions that are developed to be effective and adaptive to their contexts and systems. Exploring professional stakeholders’ views on these issues, and their knowledge of local and wider contextual factors, may therefore help to develop acceptable interventions that have stakeholder “buy-in”, are more feasible to implement and are maximally relevant to policy and practice [37].

In this context, we undertook a broad program of work that sought to conceptualize local systems of harmful commodity consumption that produce inequalities within the night-time economy [38]. As part of the broad program of work, we conducted stakeholder engagement with practitioners and academics who have an interest in health and the NTE. We formed a NLP (National Learning Partnership, NLP) with stakeholders. Through workshops and individual conversations with NLP members, we identified a focus on alcohol and gambling in the NTE. Stakeholders felt the evidence base particularly for gambling requires further progression as well as recognizing the scale of alcohol-related harms, which is a key problem in the NTE.

The present study presents findings from qualitative research in which we engaged with local authority stakeholders, in two different UK locations, with the objective of assessing their views on the harms and health inequalities in the NTE. We focused specifically on inequalities from alcohol and gambling, their relationship with the NTE, and how different forms of power might underpin the inequalities generated.

## Methods

### Study Design and Study Team Characteristics

This qualitative study involved semi-structured interviews with stakeholders who had knowledge and/or experience of the implementation of local authority activities and interventions in the NTEs of two predominantly urban local authorities in England. All members of the research team had prior experience of qualitative research. The study is reported following Standards for Reporting Qualitative Research guidelines.

### Study Population

#### Site Selection and Recruitment

The following criteria for selecting two case study sites were developed with support from the NLP: (i) proximity to the research teams for ease of access (who were based in London, Yorkshire and Humber and South West England); (ii) geographical variation to enable a comparison between these regions with reference to the UK levelling up-agenda (government policy to reduce geographical inequalities); (iii) within-area variation in socioeconomic status (based on the Index of Multiple Deprivation) [39]; (iv) the presence of a significant NTE; and (v) variation in approaches to addressing harms in the NTE (determined by scoping of local policy documents). We approached potentially eligible sites based on these criteria and obtained approval to participate through their Director for Public Health, initially through email and videoconference calls.

Swindon’s NTE is geographically split and segmented into two areas—the Old Town or Downtown[40]. This is in comparison to Derby’s NTE which is compact, allowing venues to serve different markets in the same locality which are more accessible to visitors[41]. In both Swindon and Derby, violence and sexual offenses were the most common offenses from February 2025 to January 2026, accounting for 38% of crimes in Swindon[42] and 40% in Derby[43]. Addressing alcohol-related crimes remains a key priority for both local authorities. In Swindon, the local police service reported a 34% increase in the number of people arrested for drink-driving in December 2025, relative to December 2024[44]. In Derby, violence without and/or with injury made up over half of recorded crimes in the NTE between 2020 and 2023[45].

### Participant Selection

We aimed to recruit up to 10 participants from each of the two case study areas [46]. Participants were eligible to participate if they had knowledge and/or experience of implementing local authority activities and interventions in the night-time economy, such as local authority public health staff and leaders of community and voluntary sector organizations. Professionals were identified through online searches of local council websites, through our research team and the study NLP, and through snowballing techniques, and then purposively sampled to ensure that the sample had a diverse range of roles and experience relevant to the NTE (e.g., police, public health, third sector). We sent a recruitment email to these professionals, attaching the study’s information sheet and consent form and asking them to reply if they wished to take part.

### Data Collection

Interviews took place between October 2023 and January 2024 via video conferencing (Microsoft Teams) and lasted an average of 49 min (range: 35–60 min). These were conducted by CM, PMH, KP, and ZLC and audio-recorded using the built-in videoconferencing recording function and an encrypted audio recorder after participants gave informed consent. We used a semi-structured topic guide (Appendix 1) developed by the research team to ensure key topics of interest were covered, while allowing participants to raise new issues. These topics included the following: understanding the local context, priorities for the night-time economy, initiatives (in the past five years), and barriers and facilitators to reducing health inequalities from harmful commodities in the NTE. Prompt questions included a focus on alcohol and gambling, as stakeholders we engaged with prior to this study identified these as priority issues for their respective NTEs. Participants also identified a diverse range of NTE interventions used in the local case study sites (Appendix 2).

### Ethical Considerations

Ethical approval for this study was granted by the University of Bristol’s Health Sciences Faculty Research Ethics Committee before its conduct (ref. 15,126). Ethical approval was also granted by London School of Health & Tropical Medicine (ref. 9829) and the University of Sheffield (ref. 909).

### Data Analysis

Data were analyzed following the framework method[2] (Gale et al., 2013). Interview recordings were transcribed and anonymized by a professional transcription company. The research team checked the transcripts against the original recordings to ensure accuracy and imported these into NVivo11 [47]. PMH, MPD, and CM read the interview transcripts to familiarize themselves with the data. Following data familiarization, deductive code labels, derived from McCartney et al.’s theorized sources of power framework [8] (described further below), were formulated. A data-driven framework analysis approach was chosen, a form of thematic analysis which involves using a coding theme to help identify, interpret, and describe recurring themes across a dataset[2]. The sources of power framework were developed to outline six key sources, forms, and positions of power: (i) economic, (ii) knowledge, (iii) culture and belief, (iv) collective organizations, (v) state, and (vi) positional, as well as the social spaces in which they operate. This framework emerged as particularly relevant to the aims of our study, as McCartney and colleagues propose that this framework can support identification of sources of power that maintain, influence health inequalities, and help focus action to reduce such inequalities [8]. The sources of power framework were identified in the complementary literature review as part of the wider study. Further information regarding the sources of power framework can be found in Appendix 3. These codes were applied to all relevant data excerpts. The data were charted into matrices in Microsoft Excel and paraphrased for each code with reference to notable quotations. Candidate themes were developed by identifying data patterns within and between matrices. The final themes and subthemes were reported as an analytic narrative with accompanying illustrative quotes.

## Results

### Site Characteristics

Table 1 summarizes the key characteristics of each case study site.

**Table 1:** Summary of the key characteristics of included sites

	**Swindon**	**Derby**
Location	Large town located in Wiltshire, South West of England	University city in Derbyshire, East Midlands of England
Population (2021 census)[48]	233,400	261,400
Number of households (2021 census)	95,862	105,652
Average life expectancy gap (years)*	Females: 4.4 Males: 5.7 All: 5.05	Females: 8 Males: 11 All: 9.5
Location description	Known as a major railway town, divided into a modern center and historic Old Town. Bidding for the UK City of Culture 2029, to help revitalize the town center	Known as the birthplace of the Industrial Revolution, with attractions that are part of a UNESCO World Heritage site. Known to be a major center for advanced transport manufacturing
NTE initiatives	In 2022, the Safety at Night Charter was developed by Wiltshire Police, Swindon Borough Council and Wiltshire Council to improve the safety of the NTE in Swindon and Wiltshire Other initiatives that are in operation in Swindon’s NTE include PubWatch, Ask for Angela, Street Pastors and the Walk Away Campaign (to reduce alcohol and drug-related violence in the NTE)	Derby city has had Purple Flag status for its NTE since 2013 (award for town and city centers that meet excellence standards in the NTE) Other initiatives that have been developed include the Safe Derby initiative (increasing awareness about violence towards women in public places), Street Pastors and Girls Night In Campaign (addressing increased incidents of spiking in the NTE)

*Difference between the most deprived 10% of the population and the least deprived 10% of the population, as defined by the deprivation deciles from the Index of Multiple Deprivation 2019 (Consumer Data Research Centre., 2024)

### Participant Characteristics

We interviewed 17 stakeholders (85% of invitees). Ten participants were employed within roles that were relevant to Swindon’s NTE and seven within Derby’s NTE (Table 2). The majority of participants (fourteen) worked within the local authority in various teams, including public health roles relating to health promotion (eight), policing (three), community safety (two) and planning (one). The remaining three worked in relevant third sector roles. All participants engaged with the NTE as part of their job role and had at least five years of experience. Ten had senior management or director roles.

**Table 2:** Characteristics of the interview participants

Local authority	Number of stakeholders	Participant gender	Job role seniority	Employment sector
Swindon	11	7 males 4 females	4 middle management 7 senior management	8 local authority 3 third sector
Derby	7	3 males 4 females	4 middle management 2 senior management	6 local authority 0 third sector

### Themes

We present our findings according to McCartney’s Sources of Power framework but note that these sources of power categories are not mutually exclusive. Some themes may have had relevance to multiple categories but were placed in the most relevant category for ease of presentation. Table 3 shows the themes and sub-themes developed from the data; these are the focus of the analytic narrative presented below.

**Table 3:** Thematic table of study findings, following McCartney’s Sources of Power framework [8]

Sources of power^9^	Sub-themes (with main discussion points)
Theme 1: Economic resources (income and wealth; markets; businesses)	● Austerity negatively impacting the NTE ● Cuts to public spending and funding allocation ● COVID-19's detrimental impact
Theme 2: Knowledge (lobbying; advertising; social media)	● Young people’s low knowledge of gambling harms ● Third sector organizations’ local knowledge helping to identify vulnerable people within communities
Theme 3: Culture and belief (religion; social norms)	● Altered cultural practices and behaviors within the NTE following COVID-19 ● Importance of place and identity to attract visitors to an NTE
Theme 4: Collective organizations (political parties; trade groups; charities)	● Need for collaborative, coordinated and streamlined working between different stakeholder groups ● Importance of community groups
Theme 5: State (government; military, physical, legal force)	● Role of licensing and regulations within the NTE ● Working with teams with conflicting agendas
Theme 6: Positional (hierarchies and networks)	● Deprived areas and households excluded from or more adversely affected by unhealthy commodities in the NTE ● Ethnic minority groups have unique NTE risk profiles

#### Theme 1: Economic (Income and Wealth)—Investment in NTE Regulation

The cumulative impact of years of economic decline in the NTE was recognized by participants from both Swindon and Derby. This decline was described as a combination of public and private disinvestment, coupled with the economic impact of COVID-19. Consequently, participants commonly discussed the impact of austerity measures and disinvestment affecting the NTE: *“It’s gone downhill – absolutely gone downhill”* [Swindon Third Sector P5]. Stakeholders felt that engagement from the central government in terms of financial investment and NTE regulation had the potential to benefit businesses by improving safety, quality and reputation.

Despite receiving monies from the central government, participants explained that there were stipulations on how this was spent, preventing local authorities from investing in initiatives deemed a priority at a local level. As one participant explained, *“We’re guided by the Home Office in… what we can spend the money on – so there are restrictions that we have to work towards”* [Swindon Local Authority P6]. Participants expressed frustration at the lack of flexibility in how funds could be spent to address the needs of local communities. Furthermore, regular budget cuts meant that funded NTE initiatives were often only in place for short periods of time, which hindered long-term and sustainable change.

*“We get this every time, we have some really good initiatives and then budget cuts hit, whether they hit the Police and we all get taken away, that’s what happened with the licensing officers, they all got taken away….”* [Derby, Local Authority P3].

As a longer-term consequence of austerity and funding conditions, participants felt that the deterioration of the NTE and wider town centers was a barrier to private investment in the local area. Participants also acknowledged that the type of investment that the areas needed had to be appropriate and meet the needs of the community:

“Those office to residential conversions, etc., it’s not the type of investment you want” [Swindon Local Authority SW9].

*“One of the challenges of the town centre is it’s just not getting the good quality investment that it needs”* [Swindon Local Authority P9].

This lack of investment was felt to have led to dilapidation:

*“Swindon’s town centre, it’s had a bit of a bad reputation for decades to be honest”* [Swindon Local Authority P9].

The COVID-19 pandemic was considered a catalyst of this dilapidation, particularly with respect to business investment:

*“COVID has really had a detrimental impact, businesses are leaving left right and centre and it’s not the best of places to walk through”* [Swindon Local Authority P1].

The COVID-19 pandemic was also felt to have affected local authority stakeholders’ working relationships with NTE business owners, due to staff going on furlough or changing roles to seek alternative employment outside of the NTE:

*“Straight after these months of the lockdowns we particularly found that really difficult with some of the security staff because… all of our partnerships had sort of broken down because people had moved on”* [Derby Local Authority P3]

The impact of austerity measures and COVID-19 pandemic restrictions meant that interventions developed to address inequalities in local communities were short-term and did not promote sustainable area change.

#### Theme 2: Knowledge—Understanding and Awareness of Harms

Knowledge in this study related to the general public’s understanding of harms as well as awareness about commercial determinants of health and inequalities in health outcomes. References to knowledge were mainly limited to the highlighted lack of awareness of harms and inequalities in the NTE, particularly with regards to alcohol and gambling.

Participants highlighted a “*lack of awareness of gambling harms in young people*” [Derby Local Authority P1]. While 18 years is the minimum legal age for gambling in the UK, participants noted *“the ease of access among young people…but actually through our focus groups they’re quite aware of gambling”* [Derby Local Authority P1]. Although this was discussed by several participants in each LA, Derby participants more strongly emphasized this lack of awareness.

Participants discussed the need for other stakeholders, for example, NHS workers, to recognize the wider impact that addictive behaviors such as gambling can have on health outcomes:

*“But helping the NHS to see that actually gambling, it fits with these other things, these other addictions, it has these impacts on health and start to see the importance of it as a factor in people’s health outcomes”* [Derby Local Authority P2].

Substance misuse charities were noted as being key to identifying individuals at risk of adverse lifestyle harm and known hotspot locations:

“*the bus station was an area in (town) that was named as an ‘area of problem’ with people gathering, drinking, taking drugs*” [Swindon Third Sector P5].

Participants noted the need for charities and other third sector organizations to help local authority areas to engage with communities deemed to be at higher risk of harm. Their local knowledge and connections would be important to integrate into future work, particularly those that work at a grass-roots level.

#### Theme 3: Culture and Belief—How Communities Engage with the NTE

Participants reported that users of the NTE have altered their behavioral patterns with respect to the NTE in the past few years, away from regular nights out in the town center. Participants recognized that this could have been exacerbated by the COVID-19 pandemic and subsequent cost of living crisis. It was also felt that the general public were moving away from the NTE in the town and city centers and choosing to engage with their neighborhood NTE instead. Participants felt that as a consequence, there was concern about harms taking place in these neighborhoods where there were fewer tools available to influence and/or reduce harms.

*“Numbers visiting are down, taxi numbers are down. Street pastors interactions are down… that 7 till midnight audience are staying more local post COVID. They’ve found the local pub that actually has a fairly decent offer…”* [Derby Local Authority P1].

Participants highlighted that a sense of place and identity is an important factor in attracting visitors to an NTE. Participants recognized that certain parts of Swindon’s NTE lack character and individuality, limiting the appeal of the NTE and town center to residents and users:

*“…it [town centre] doesn’t have many, for example, older buildings. It doesn’t have a river running through it. It doesn’t have a public space. It doesn’t have that character, that identity, that sense of place”* [Swindon Local Authority P9].

The cost-of-living crisis and a lack of character were cited as discouraging local residents from using the NTE. It was suggested that future policies needed to recognize these to help inform appropriate strategies.

#### Theme 4: Collective Organizations—Adoption of a Whole-System Approach

The need for collaborative, coordinated, and streamlined working between different stakeholder groups was identified as a high priority for both local authorities. Despite the challenges of funding and local-level issues, participants universally agreed on the importance of collaborative working:

*“… the community support officers, those public protection officers within the council work really, really closely with the police, so they have that really good sharing of local knowledge”* [Derby Local Authority P1].

Participants also discussed situations where this coordinated working was absent and stakeholders had conflicting agendas, making challenges harder to resolve:

*“…you have regulatory services, so licensing, trading standards, all sitting together but then you’ve got your community safety aspect, all completely different, all in completely different departments and none of the two will talk to each other. That can be really difficult”* [Derby Local Authority P3].

Continued collaboration and the adoption of a whole-system approach were suggested ways of reducing harms in the town centers and users’ perceived safety.

*“I think from our harm reduction approach certainly it’s potentially joining up with other services that are operating in a night time economy like your door staff…. your taxi marshals and perhaps having specialist drugs workers and alcohol and mental health workers working out of a control room in the town centre and being able to respond to potential concerns…”* [Swindon Local Authority P4].

As part of a whole-system approach, participants wanted to continue engaging with local businesses, through initiatives such as PubWatch [49]. However, the cessation of the local authorities’ Business Improvement Districts (BIDs) due to a lack of funding made this more challenging and participants were concerned about the longevity of collaboration:

*“….they* [Business Improvement District] *didn’t have enough money. Really sad… they had a lot of ambition, but if you haven’t got the money behind you, you can’t really do very much”* [Swindon Local Authority P1].

Participants commonly spoke of the importance of engaging with local “*community groups and faith groups”* [P2 & P3] to improve public health and build trusting relationships. These relationships were considered crucial in creating a proactive rather than reactive approach to local needs and issues:

*“…it’s all about trying to reduce the harm within the communities that are that kind of primary, secondary stage before it gets to the tertiary stage – and will need quite significant intervention”* [Swindon Local Authority P6&P7].

While collaboration and engagement were identified as key priorities to participants, it was recognized that any future interventions must be accessible to community groups and local businesses and developed in conjunction with them.

#### Theme 5: State—Licensing and Regulation Within the NTE

Participants offered examples of state powers (relating to legal, regulatory and public services) operating within the NTE, including licensing visits: “*To see them [licenced venues] in action and just check that they are complying with their licenses. We do those visits often in conjunction with the police…”* [Swindon Local Authority P1]. While they recognized that there was often a negative connotation associated with these visits, participants felt it gave them a good opportunity to engage with business owners routinely and face-to-face.

Community initiatives such as PubWatch were also promoted by local authority departments to encourage regular communication not only between public houses, licensing teams and the police, but also other business owners to help identify any local issues and possible solutions. Alongside multi-agency engagement, increasing police presence at peak times gave stakeholders the opportunity to proactively address issues in the NTE. *“On a Friday and a Saturday night they’re [police] visibly present, they’re engaging with the community and they’re dealing with issues very openly if they arise.”* [Swindon Local Authority P1].

There were concerns among participants that the 2003 Licensing Act [50] was outdated and prioritized the economic benefits of alcohol licenses over their potential harms:

*“…I think it’s time that [the] Licensing Act got a big update. I would personally like to see a fifth licensing objective of health because I just don’t think that’s included whatsoever….”* [Derby Local Authority P1].

Participants discussed the challenges of working with different teams within the local authority, whose work does not include public health and health as key priorities. As a consequence, participants reported that public health impacts were not consistently considered when reviewing licensing applications: “*It [licensing decision] was very much from a licensing and location-based planning kind of thing rather than any consideration about the health harms”* [Derby Local Authority P1]. The inconsistencies in how licensing applications are approved within local authorities meant that participants felt that the NTE users were not necessarily considered in regard to licensing and planning application and the potential health harms. At the same time, participants recognized the challenges of encouraging businesses to operate in the NTE and the need to support applications to open premises to benefit the local economy.

#### Theme 6: Positional—Accommodating for Different Communities in the NTE

Positional power relates to the networks that operate within the NTE that can facilitate discrimination, stigma and influence via exploitation of positional advantage, leading to unequal health outcomes. Participants highlighted areas of deprivation that surround the main center of their locality and the different ways that individuals from these areas access the NTE. NTE users often preferred to engage with their local neighborhood NTE as this was more accessible to them. Through local authority stakeholders *“always focusing on the city centre…missing the district centres”* [Derby Local Authority P2], the impact of deprivation on health and wellbeing was often missed. Participants recognized that more consideration needed to be given to how people from low-income households were using the NTE alongside being more at risk of experiencing crime in these spaces:

*“…a couple of weeks ago where there was a female who was out and these group of men were saying they were a taxi and tried to get her in a taxi and it came out that she had been sexually assaulted by these males…. I do wonder if that comes into that socioeconomic space because actually if someone is lower on funds and they are offering a cheaper fare are they likely to get into that taxi”* [Swindon Third Sector P5].

Strategies to address violence against women and girls were identified as priorities in both local authorities, particularly in light of historical gender-based crimes in the areas:

*“This [taxi safety campaign] was funded through the ‘Violence Against Women and Girls’ agenda from the Home Office…. because of the murder… women have got a fear of being on their own in taxis"* [Swindon Third Sector P5].

Participants highlighted a need for NTE spaces to accommodate and protect all members of the different communities they serve. Subgroups considered to have unique but unmet support needs and concerns in the NTE included men, university students and minoritized ethnic groups:

*“You’ve got your white British males. I would say they’re potentially the ones that are going out binge drinking in the night time economy, and potentially your weekend powder cocaine users. I guess they’re at increased risk of the impact on their health…”* [Swindon Local Authority P4].

“*They [South Asian ethnic minority groups] do drink at increased levels but we don’t really know where they’re drinking. They’re certainly not really engaging in core services either”* [Swindon Local Authority P4].

Participants also highlighted an increase in hate crimes and reluctance to report crimes taking place in the NTE, particularly among the LGBTQ+ (lesbian, gay, bisexual, transgender, or queer—LGBTQ+) community, resulting in them disengaging from the NTE:

*“[LGBT*+ *people are] Being spat at, being shouted at, being abused – particularly on public transport”* [Swindon Third Sector P5].

*“the majority of [LGBTQ*+*] people we spoke to said they don’t go out after dark”* [Swindon Third Sector P5].

Participants who had engaged with the LGBTQ+ community spoke about how those who had experienced hate crimes were reluctant to report incidents to the police *“because they didn’t think they’d be taken seriously – they didn’t think the police were that interested in it. They thought they were under-resourced, so they’d have better things to do”* [Swindon Third Sector P5].

It was highlighted that each population subgroup has different support needs and risk profiles and these need to be addressed to encourage them to benefit from the NTE or be protected from potential harms.

## Discussion

This study analyzed professional stakeholders’ views on systems of consumption of unhealthy commodities in the NTE, particularly alcohol and gambling, the health inequalities generated as a result, and the priorities and challenges associated with these issues. McCartney et al.’s (2020) power framework helped to summarize the sources of power that shape systems of consumption, leading to unequal health outcomes.

In the first theme relating to economic power, stakeholders discussed the impacts of austerity measures and disinvestment, particularly following the COVID-19 pandemic, combined with a lack of power on how limited funding could be spent, which they perceived to lead to a deterioration in the NTE. They were receptive to greater investment and regulation to help support NTE businesses and improve safety, quality and reputation. Knowledge, the second theme, focused on the lack of awareness among NTE users and the wider general population regarding existing harms and inequalities in the NTE, particularly regarding alcohol and gambling and how these harms and inequalities can lead to wider adverse health outcomes. Local authority participants described the work that the community sector does as valuable, and how to best utilize them to enable statutory connections to more vulnerable groups in the population. The third theme, culture and belief, related to changes in how the local community uses and engages with the NTE, particularly as a result of the cost-of-living crisis and COVID-19 pandemic. Participants stated the importance of encouraging places to have an identity to enable safer and more enjoyable use of the NTE.

The need for collaborative working between different stakeholder groups was identified as a high priority in the fourth theme, collective organizations. Based on the perspectives of stakeholders from both local authorities, adoption of a whole system approach to NTE management might help local authorities focus efforts on reconciling conflicting agendas and delivering more cohesive policies. Furthermore, engaging with community groups to help develop a whole system approach that is accessible to wider groups of the population was considered important. The fifth theme, state power, showed that the licensing visits and community initiatives were promoted by local authority departments, despite negative connotations associated with them. The challenges of working with different teams who do not always prioritize public health was also highlighted. The sixth and final theme, positional power, reported that NTE users often prefer to engage with their local neighborhood NTE as it is considered more accessible but that these places may be less well regulated. Participants highlighted a need for NTE spaces to accommodate and protect all members of the different communities that they serve.

The findings reported in this study were developed using the sources of power framework, to help inform policy makers of health inequalities exacerbated by different forms of power within the NTE. The sources of power framework, developed by McCartney et al. [8] served as a lens to present the stakeholder views about the causes of health inequalities in the NTE. While structural inequalities are routinely acknowledged to cause health inequalities, the specific ways in which they impact harms from gambling and alcohol are less well documented [51, 52]. Participants noted that financial constraints led to difficult prioritization decisions for the NTE, including funding cuts for various initiatives, potentially impacting the most vulnerable members of the local community. Social conditions have been previously identified as the fundamental causes of health inequalities, however, to address this, stakeholders must recognize the impact of power on reducing social and economic inequalities together as a united multi-agency [53]. The sources of power framework provide us with the opportunity to understand how these health inequalities within the NTEs of two local authorities arise and perpetuate.

Stakeholders identified that different population sub-groups had differential experiences in using the NTE in their local authorities and consequently had different support needs. Furthermore, it suggests the stakeholders’ sense of inability to orientate services or interventions according to local specific needs and differential needs of different populations. In particular, participants reported that the LGBTQ+ community spoke about crime incidents and feeling unsafe within the NTE, while white males were identified by participants as being most likely to engage in excessive drinking, as documented elsewhere [54–58]. Previous work has found that increased police presence and targeted interventions in areas with greater alcohol-related violence are more effective, particularly in multi-agency collaboration [59], as reported by participants in this study. Participants acknowledged that several population sub-groups were underserved in terms of engagement and opportunities to provide them with protection and support as appropriate. Similar challenges have been documented elsewhere [60–62], particularly for some ethnic minority communities among which home drinking may be more common [63–65]. The Stockholm prevents Alcohol and Drug problems (STAD) approach was developed to restrict the availability of alcohol in four environments, including home settings [66]. Consequently, the Drink Less, Enjoy More intervention was developed on the foundations of the STAD approach to address binge drinking in the home setting within Wrexham [66]. Components of these interventions, including tailoring messages through existing policies and legislation to specifically deter excessive drinking, could be relevant to addressing issues surrounding excessive home-based alcohol consumption, prior to going out to licensed establishments (a practice known as pre-loading), in Swindon and Derby.

### Implications for Policy, Practice, and Future Research

Our findings highlight complexities for interventions aiming to address health inequalities that arise in the NTE, particularly in the context of multi-agency working and conflicting stakeholder priorities. Participants identified that their local authority had distinct public health and economic priorities, which sometimes clashed. This could be seen in their descriptions of how positive benefits for the local economy were sometimes prioritized over negative impacts on local public health when reviewing alcohol license applications. Participants in this study acknowledged that the lack of power held by local authorities in how limited funds are spent to address local community-specific needs hindered progress and therefore, conflicting priorities continued to exist. There was also agreement across the board that in order to address these issues, long-term collaboration and the adoption of a whole-system approach should be considered to help reduce harms in the NTE.

Alongside the need to address conflicting stakeholder priorities, participants discussed the influence that funding constraints can have on addressing NTE harms. Where adult and children’s social care, and other statutory duties, now consume over two-thirds of councils’ budgets, this leaves minimal funding for non-statutory functions, like the NTE[67]. Within the UK Government’s post-legislative scrutiny, evidence found that “underfunded local councils could not be expected to support and promote the late-night economy” [68]. This suggests that increased funding is needed to ensure that local authorities are able to prioritize addressing harms and health inequalities in the NTE, rather than simply relying on the financial revenue that the NTE generates to plug these funding gaps. Furthermore, the most deprived local authorities were worst affected by central government spending cuts, with a 33% reduction in spending power versus 14% in the least deprived areas between 2010/2011 and 2019/2020. NTE users often preferred to engage with their local neighborhood NTE as this was more accessible to them. However, it was noted that NTE interventions *“always focus[ed] on the city centre…missing the district centres”* [Derby Local Authority P2]. This was felt to have implications for health inequalities, as NTE users in less affluent districts are accordingly more exposed to the impacts of deprivation on, for instance, safety in the NTE.

The relationship between health and economy has been well documented [69–71]. One of the main barriers is the perception that prioritizing economic growth within communities will automatically lead to improvements in public health [72]. Modi et al. (2022) discussed that in order to prioritize health, cross-government accountability is needed and senior level buy-in is important to ensure that a ‘health across all policies’ approach is followed [72]. Participants in this study discussed the opportunity for the 2003 Licensing Act to be updated to include a consideration of health harms and outcomes related to alcohol. A report by Audit Wales supported our stakeholders’ opinions that new and integrative collaborations for strategy development are needed to protect and improve community services, particularly when future funding is unpredictable [73]. This conflict is also emphasized by central government efforts to encourage local authorities to develop public-private partnerships to help reduce expenditure and simultaneously improve service levels [73]. Over the last decade, there has been an increase in commercial activity among local governments as a way of addressing the real-term reduction in available funding [68].

Future research should continue to engage with stakeholders and communities to develop a more comprehensive strategy to address the health inequalities that exist within the NTE. Alongside this, more consideration needs to be given to how people from low-income households and minoritized groups engage with and are protected from harms in the NTE, as our findings suggest that a range of groups is made vulnerable to greater harm by the unequal deployment of different sources of power (such as economic and knowledge). Further engagement with other local authorities would help to understand the prevalence of these health inequalities in other NTEs and how a strategy can be used by local authorities in various UK locations.

### Strengths and Limitations

This is one of the few qualitative studies to explore professional stakeholders’ views and experiences of systems of consumption of unhealthy commodities in the NTE and the harms and inequalities this can generate, thereby offering novel insights to help identify potential strategies that local authorities can use to address these harms and inequalities.

Our participants were recruited from two local authority areas in the UK and purposive sampling was facilitated by participant existing networks. While this sampling technique meant that we were able to interview participants that had a remit in the NTE, it may also mean that we interviewed those who are particularly invested in prioritizing action in the NTE. It is also important to acknowledge that the research was limited to the perspectives of professional stakeholders and did not include the perspectives of NTE users, residents, and workers; future research should explore their views and priorities. Furthermore, in one local authority area we had limited representation from the third sector, which may have affected the findings had their perspectives differed from those working within the local authority.

While our findings suggest that the main themes found in this study are similar across the two local authority areas, i.e., that NTE harms are unequal and local authorities have experienced financial cuts, our findings need to be interpreted with an awareness of the specific characteristics of the two case-study areas included. Engagement with other local authority areas, building on the findings of this study, would offer a useful insight into the views of a wider range of stakeholders.

## Conclusion

This study highlights the conflicting priorities within local authorities between public health and potential economic opportunities in the NTE. Stakeholders who have a remit in the NTE need to ensure that their work aligns with other areas and strategies are developed in consideration of wider challenges that could influence the NTE. Findings from this study can be used to help policymakers inform their decision-making process as this is one of the few qualitative studies that explored multiple commodities associated with health inequalities in the NTE.

## Data Availability

The data that support the findings of this study are available on request from the corresponding author. The data are not publicly available due to privacy or ethical restrictions.

## Appendix 1: Semi-structured topic guide used in South West Local Authority and East Midlands Local Authority interviews

**Introduction:**

- Thank you for participating
- Introduce self and NIHR SPHR (funder)
- Introduce the study
- Talk through key points:
  • length of interview
  • interview like a discussion, but will cover key topics
  • no right or wrong answers
  • participation is voluntary, rights to withdraw
  • recording interview (concentrate on what you are saying, accuracy)
- Confidentiality and anonymity, how findings will be reported
- Questions?
- Happy to proceed? Sign consent form

**Start recording**

**1. Participant background/role**

- Clarify current role and responsibilities
- Organisation (purpose; main activities)
- Role and responsibilities in relation to the night-time economy

**2. Understanding the local context**

- What are the key challenges in relation to the night-time economy (NTE) here?
  o Issues relating to Alcohol, tobacco, gambling, high-fat-salt-sugar foods?
  o Inequalities in health relating to the NTE?
- Are there other challenges relating to harmful products – outside of the NTE?
  o Alcohol, tobacco, gambling, high-fat-salt-sugar foods?
  o Inequalities in health relating to these?
- Description of the local authority area and the drivers of affects the above issues:
  • Socio demographics
  • Geography
  • Key employers/business/economy
  • LA budget/funds
  • Local democratic structures

**3. Priorities for the night-time economy**

- What are the key local priorities in relation to the NTE?
  • Your personal priorities/organisational priorities/others’ priorities
  • Are there separate priorities relating to unhealthy products outside the NTE—Alcohol, tobacco, gambling, high-fat-salt-sugar foods?
  • How do these policies link (between daytime and night-time economies for example?)
- Key stakeholders on the local night-time economy – who is engaged? Who is ‘missing’ from work to reduce inequalities in the NTE?
  • LA
  • CCG
  • Other statutory agencies
  • Third sector partners
  • Elected members
  • Private sector
  • Residents
  • Others?
- Who are the key decision makers?
- Who should be involved but is not?
- Describe the process through which LA priorities for the night-time economy are determined
  • Involvement in this
  • Use of evidence
  • Advantages/disadvantages of local processes
  • Any unique factors in this locality

**4. Initiatives (in the past 5 years)**

- What have been the key local policies to improve the NTE and/or health issues in the NTE
- Other policies on unhealthy products (that are unrelated to the NTE?)
  o Alcohol, tobacco, gambling, high-fat-salt-sugar foods?
- What have been the key interventions (if different)
- How have these been funded (and changes to funding, constraints etc.)
- Success or otherwise of these (including on health inequalities)
- Planned policy/initiatives
- What policy/initiative would respondent most like to see
  • What prevents this?

**5. Barriers/facilitators to reducing inequalities in health from harmful products in the NTE**

What are the key barriers to reducing health inequalities in this context? Can you provide some examples of work that has stalled/failed to get off the ground?

- Financial
- Prioritisation
- Evidence
- Policy/practice change
- Limits on capacity to influence key determinants
- Stakeholder resistance
- Legal limits/threat of legal action
- Other

What has enabled successful work in this area?

**8. Thank and close**

- Ask if anything else they'd like to add
- Any suggestions for other participants to take part (and can they facilitate introduction)
- Any key documentation that would be useful to share with study team
- Thank and close

## Appendix 2

Table 4

**Table 4:** Interventions identified in South West Local Authority and East Midlands Local Authority interviews

Alcohol and substance abuse • Brief alcohol interventions (pharmacists) • Alcohol and substance misuse services (referral) • Challenge 18 • Multi-agency response to tackle under-age drinkers • Drink driving campaigns • Super Swindon Outreach Service (multidisciplinary misuse intervention)	Policing and crime • CCTV in vans • Cardiff data reporting • Clear, Hold, Build • Fast food outlets offering free meals for forfeiting knives • Plain clothes police patrols • Serious violence duty • Police power to direct first-time offenders to alcohol and drug misuse services • Safety sweeps to remove weapons and drug paraphernalia
Local authority policies • Community Safety Partnerships • Local Authority alcohol strategy • Local Authority and police response to drink spiking • Public Space Protection Order to penalize and prevent car meets • Regulatory Services to prevent illegal tobacco sales	Training and community groups • Bystander training • Door staff training • Taxi driver language communication • Street pastors • Taxi marshals • Safer neighbourhood team • Sex work support workers • Goody bags for NTE users
Environment Purple Flag Scheme Safer Streets	Businesses and licencing • Licensing SAVI scheme • PubWatch • Cumulative impact policy to restrict new alcohol licenses
Violence against Women and Girls • Ask for Angela • Drink toppers (bars, pubs, clubs)	Gambling NHS gambling harm service

## Appendix 3: Sources of power theoretical framework (McCartney et al., 2021)

The framework was developed by McCartney and colleagues to identify important sources of power, and the environments in which they operate, alongside the positions and forms of power relationship involved [7]. It was considered appropriate and adapted for this piece of work, as it provides the opportunity for users (e.g., policymakers, practitioners and academics) to use the framework in a variety of contexts to help identify existing issues and areas for future research.

### Economic resources

The first source of power in the framework is economic (income and wealth). This relates to the power derived from the income and wealth that people have, arising through markets for goods, services and labour, and being differentially paid for labour. Economic power can take form in a variety of ways and contexts. Typically, whoever has ownership of economic assets has the means to exercise economic power and the opportunity to exploit and/or dominate labour. However, individuals who may have less income and wealth may also be able to generate economic power through collection action and decision-making through employment strikes and disputes, for example.

### Knowledge

The second source of power in the framework is knowledge, which is split into three categories: generation, media (communication) and the education system. Power emerging from knowledge generation comes from academia and government, alongside lobby and/or campaign groups and charities. Knowledge generation is closely related to knowledge communication and the different types of media outlets used, for example, broadcasting, internet, print). Linked to economic power is the ownership and control of knowledge communication through the control of release of knowledge and information, framing public conversation and influencing opinions. Knowledge through the education system includes formal settings such as, nurseries, schools and universities, but also among families, workplaces and the internet. The internet has become a more influential source of power, and the use of specific and personalized advertising should be recognized. Those who are involved in the decision making of curriculum content and funding, teaching in these settings alongside having specialized knowledge are part of this form of knowledge power.

### Culture and belief

The third source of power in the framework is culture and belief. This refers to the context of social norms and the shared values and understandings among populations alongside organized religions. Those in positions of power include religious leaders and those with formally developed social networks and outreach. Use of power in this context can include influence social norms and values, influencing networks and communities alongside social relations (for example, stigma, discrimination and exclusion).

### Collective organisations

The fourth source of power in the framework is collective organisations. This includes political institutions, for example, political parties and parliament, and other collectives, for example, trade unions and charities. There are opportunities for those working in this space to confer with power including elected representatives, voters and activists.

### State

The fifth source of power in the framework is the state. This refers to both through the government and military, using physical and/or legal force. Whilst government power relates to legal, regulatory and public service, the armed forces, policing and custodial spaces relates to military, physical and legal force. This involves individuals who act as elected officials, military leaders, the judiciary and the police.

### Positional

The sixth source of power in the framework is positional. This refers to the hierarchies and networks that operate, including workplaces, schools, domestic relationships and relations between gender. The positional source of power can manifest in several ways including discrimination, stigma, social networks and influence.
